# Adaptive emotion-aware chatbot for mental health diagnosis using recurrent reinforcement learning and transformer models

**DOI:** 10.3389/frai.2026.1769286

**Published:** 2026-05-05

**Authors:** Sonia Dessai, Sandhya Arora, Gayatri Joshi

**Affiliations:** 1 Department of Computer Engineering, MKSSS's Cummins College of Engineering for Women, Pune, India; 2 Association for Computing Machinery's Council on Women in Computing (ACM-W), New York, NY, United States

**Keywords:** chatbot, deep learning, dynamic questionnaires, Long Short-Term Memory (LSTM), mental health, proximal policy optimization, recurrent RL models, reinforcement learning

## Abstract

In the busy and stressful modern world, people tend to disregard mental health, still it is an important factor of overall health. The constant pressure to achieve success, the invasive nature of technology, and the constantly growing needs of the contemporary world may all be the causes of stress, anxiety, and other mental health difficulties. Despite growing awareness, mental health remains a sensitive topic, as social stigma and other factors continue to hinder open discourse. There are many standard tests like GAD-7 for anxiety, PHQ-9 for depression, PSS-10 for stress, and many more openly available on the internet, but people might miss estimate their situation while answering these questionnaires, leading to wrong or inaccurate diagnoses. This paper focuses on integrating the questions of these three standard questionnaires and creating an emotion-aware chatbot with a dynamic questionnaire. To evaluate user responses, i.e., to measure the severity of each mental disorder, a fine-tuned RoBERTa model is used. This fine-tuned transformer model will take in the user response and return a severity value for each of the three disorders on a scale of 0–100. The ROC curve method is applied to the MHP data-set to determine the threshold score for each question. Using the above two, a Recurrent Reinforcement Learning model is trained, which combines Proximal Policy Optimization (PPO) and Long Short-Term Memory (LSTM) to create the dynamic questionnaire. The Recurrent RL model will be trained to understand and evaluate the scores obtained from the user response and the history of the current session and dynamically decide which question to ask next.

## 1 Introduction

Most of the psychological assessments use ordinal questionnaires. Here, individual responses are assigned a numeric score that sums up to a total score, which shows the severity of a mental health condition. While these tools, like for instance, GAD-7 for anxiety, PHQ-9 for depression, and PSS-10 for stress, may offer a structured means to quantify the psychological states, they often have limitations. Respondents may find these questionnaires overwhelming, and the fixed set of questions may not capture the nuances of each individual's emotional experience. These tests are heavily dependent on self-assessment, which puts them at risk of biases, such as underreporting of symptoms due to shame or unawareness. Also, these assessments are static in nature, i.e., the number of questions in the assessment questionnaire is fixed, which limits their adaptability, as all questions will be asked irrespective of the respondent's current mental state or the relevance of the particular question to their specific mental condition. Offline monitoring of such tests and the necessity to go to different platforms or healthcare environments to receive a full and comprehensive assessment might be inconvenient and potentially contribute even more to the problem if access to mental health insights is not given in a timely manner. Some transient factors like stress, fatigue, or cultural differences can also influence the results of psychological tests which can affect reliability.

Given these problems, an emotion-aware chat application that employs dynamic and adaptive questioning offers a promising alternative, which can diagnose mental health conditions more efficiently and more intuitively. By using natural language inputs and reinforcement learning models with recurrent neural networks (LSTM), a system can be developed that can adjust the flow of questions in real-time based on previous user responses. The given method will not only decrease the workload because irrelevant or redundant questions will be minimized but also will allow understanding the emotional and mental state of users better thanks to the analysis of texts. In this manner, the user will not only find someone to open up about how they feel but will not even need to browse to other websites and take various tests to know what issue they are experiencing. Also, in this manner the user will not be required to answer all the questions, as the model will not ask the questions that do not appear to be relevant to the mental health issue of the respondent. Answering every question, this text may be a deep dive into the mental state of a patient and at the same time allow seeing the usual behavioral patterns of the person in his/her state of being down. Mental health assessment becomes more user-friendly, efficient, and accessible due to the possibility of diagnosing a variety of disorders during the same interaction.

This change to interactive, context-sensitive diagnostics (as opposed to rigid and ordinal) makes use of machine learning and natural language to better address the subjective and dynamic nature of mental health. It can benefit both clinicians and patients because it can enable them to obtain timely personalized assessments and minimize the barriers that are linked to conventional tests.

## 2 Our contribution

The recent improvements in psychology and medicine have allowed automated systems to assist in mental health assessment. However, most of the existing approaches are dependent on fixed questionnaires, which can be inefficient and often lead to patient fatigue. To address this problem, we look into how reinforcement learning can be used to dynamically select the most informative and relevant questions based on the user responses, thereby reducing the number of questions asked while maintaining diagnostic accuracy.

The aim of this paper can be summarized as:

- Eliminating the need to ask ordinal questions by replacing them with text-based sentiment analysis.
- Formally solving the problem of adaptive mental health assessment in order to avoid redundant questioning.
- A reinforcement learning-based framework is proposed that selects the next question in real time, conditioned on prior responses.

## 3 Related work

The measurement of mental health has been a key area of study in many areas, such as psychology, psychiatry, and now even computer science. These measurements traditionally used to depend heavily on the counselor examining the patient. Technology has opened it up to the world at the same time. Advances in technology have opened the study in these fields using different innovative methods, allowing for more dynamic and efficient measurements and betterment.

GAD-7 (Spitzer et al., 2006) and PHQ-9 (Kroenke et al., 2001) developed by Robert Spitzer, Kurt Kroenke, and Janet Williams, and PSS-10 (Cohen and Williamson, 1983) developed by Sheldon Cohens, have simplified mental diagnosis. Being available on the internet, they have facilitated early self-diagnosis of mental conditions such as anxiety, depression, and stress. They are ordinal questionnaires whose every question is scored with a number based on the user's answer, accumulating to a total that indicates the severity of the mental disorder. These questionnaires have become extremely popular as a result of their easy-to-use, simple nature, and fast, accurate calculations. Paper (Spitzer et al. 2006) carried out research where patients were required to fill the GAD-7 questionnaire and were interviewed by mental health professionals. The findings indicated high sensitivity and specificity, meaning that it accurately identified most of the true cases and ruled out most of the false cases. The same study was reported by Paper (Levis et al., 2019), in which volunteers had to complete the PHQ-9 questionnaires and an interview. This test also indicated that PHQ-9 has been consistent in screening depression. However, there are still some issues as it is ordinal in nature. These involve people making incorrect estimations of their state and therefore making the wrong response choices. Secondly, just selecting a choice cannot reveal the real feeling the respondent is experiencing. In an attempt to fight this problem, researchers and academics are always seeking ways of integrating technology in mental health assessment to enable diagnoses to be performed fast and correctly.

(Torous et al. 2021) illustrates the transformation of mental healthcare by digital technologies, such as smartphone apps, social media, and chatbots, as well as virtual reality. The paper identifies the ways in which these tools can increase access and enable real-time monitoring and identifies issues with user engagement, privacy concerns, regulatory issues, and the necessity of more powerful clinical evidences.

The article (Grimm et al. 2022) provides a technological solution of applying the use of GAD-7 and PHQ-9 questionnaires. They suggest that GAD-V and PHQ-V be used that uses the PHQ-9 and GAD-7 tests with open-ended questions in form of video. The other source of information (other than the questionnaire) used to determine the risk of illness is the use of video responses used to examine the language, audio, and facial features. This app can, at times, detect the presence of anxiety and depression, and which is not detected by the ordinal questionnaires mentioned above. The models however, may bring about privacy concerns to the user and not all users may be comfortable with their audio and video being taped.

In (Haque and Rubya 2023), 10 apps of mental health chatbots were studied based on their features and more than 6,000 user reviews. The chatbots provide conversational support, 24/7, and based on AI, providing judgment-free zones to people. Human-like interactions were appreciated by users, but there are problems of inappropriate responses, likelihood of over-attachment substituting actual social interaction, privacy, and inability to handle mental health emergencies. The authors suggest that it is necessary to make chatbots more effective by means of a more advanced customization, safety, and ethical design.

The article (Lecomte et al. 2020) conducts a review of 34 articles about mHealth application based on core CBT methods in adult mental health. It concludes that applications carry out cognitive restructuring, behavioral activation, and problem-solving but not exposure. The review mentions areas of gaps, such as a scarcity of rigorous trials, theory integration, and lack of transparency on the effectiveness of apps, with the need to have evidence-based, user-centered mental health apps. Several shortened versions of the PHQ-9 have also been suggested over the years including PHQ-2 and PHQ-DEP-4. These could compensate most of the deficiencies of the PHQ-9 but they have a disadvantage of being static. Also, fewer questions can be ineffective in diagnosing the patient.

To address this, paper (Abdulhussein and van de Ven 2024) suggests a dynamic approach to reducing the PHQ-9 questionnaire items of depression by premature termination according to the cumulative score of the individual question. The score thresholds are optimized through a grid search to maximize the Youden index using the NHANES dataset with 44,749 records. This method minimizes the questions to be asked to an average of 3.5 and has high accuracy and is better than fixed-item short questionnaires such as PHQ-2 and PHQ-DEP-4.

Another approach to dynamic questionnaires that is discussed in (Patilea and Stefanescu 2024) is the RLHR Framework. It makes use of deep reinforcement learning (A2C algorithm) to dynamically modify the questionnaires. It chooses questions depending on the previous responses obtained by users and hence gives the users a unique experience. The system explains the characteristics of people through statistical techniques and rectifies the biases of the answers with time. It also gets feedback on the effectiveness of its prediction of profiles to gain insights on the best questions to choose from. It also encourages generation of synthetic data during training in the event of a reduced amount of available data.

The article (Aryal et al. 2020) explains how machine learning, in this case, Support Vector Machines and deep neural networks, can be used on brain imaging and physician notes on the electronic health records to enhance the accurate and early diagnosis of a psychological disorder. The integration of both structured imaging data and unstructured clinical text and expert input reduces misdiagnosis. Some pitfalls are overlap of symptoms, data privacy concerns, and delayed diagnosis. The methodology shows great precision (as high as 98 percent) and promises better patient outcomes through integrated human-machine analysis.

The article (Lutz et al. 2022) highlights the application of systematic, therapy-based outcome measures to psychotherapy to optimize personalization and effectiveness of treatment. It recommends the shift to go beyond clinical judgment to continuous feedback and decision aids so that therapists can customize the interventions and enhance patient outcomes by using accurate and continuous monitoring.

The new developments in AI-based mental health assessments have attracted a lot of light and have helped to alleviate the load of the conventional evaluations in the process generating ethical issues of profound importance. The article (Gibbons et al. 2016) established that computerized adaptive testing (CAT) which exploits the item response theory in item banks with considerable numbers of items such as the Mood and Anxiety Spectrum Scales attain high measurement accuracy with much less questions to increase discriminant validity of mood and anxiety disorders. Based on this, (Gibbons et al. 2008) developed the method with multidimensional item response theory (IRT) and random forests to perform computerized adaptive diagnosis and testing (CADT), which can match clinician interviews on depression in less than a minute with only four targeted items on average. The manuscript, (Graham et al. 2019) discusses original research, methodology, and theory in clinical, biomedical, social, and health services sciences to develop primary care, including systematic reviews and reflection essays by clinicians, patients, and policymakers.

Literature on LLM-based and conversational AI in mental health reveals an incremental improvement starting with a (Fitzpatrick et al., 2017) systematic review assessing the effectiveness of mobile applications in the availability of interventions to depression and anxiety. This is supplemented by the (Inkster et al. 2018) research on the Wysa AI conversational agent, which also reported that the high-engagement users who had self-reported depression symptoms had significant improvements on mood. The (Xu et al. 2024) is more recent and explores the innovations in human-computer interaction in digital health with a focus on design and AI integration approaches to improve the user experience in these systems.

Bayesian Adaptive Testing (CAT), a testing method that uses Bayesian inference to dynamically adapt the items to the respondent based on his or her ability has demonstrated significant potential in mental health assessment as it improves efficiency without impairing the measurement accuracy. (Choi et al. 2010) revealed that short forms (both static and computer-adaptive) were more efficient than full-length measures of depressive symptoms, and had similar levels of reliability with much fewer items. Complementing this, (Smits et al. 2011) presented a simulation study using CES-D depression scale with CAT that proved the capability to shorten the length of the test by more than 50 percent without significantly decreasing the accuracy of the scales in estimating the severity of symptoms, which supports the idea of scalability of CAT in mental health screening.

The field of Natural Language Processing (NLP) and sentiment analysis has transformed the mental health recognition method by studying linguistic patterns in textual and social media by detecting signs of depression and other mental health conditions. It was first attempted by (De Choudhury et al. 2013) who forecasted depression based on social media posts using characteristics such as posting frequency and sentiment as a predictive factor of online behavior and clinical depression based on the various scores. Developing on this, (Coppersmith et al. 2014) measured the mental health indicators within twitter data, differentiating disorders such as depression and PTSD by means of typical language clues and use of language patterns. More recently, (Ji et al. 2022) proposed a pre-trained language model, MentalBERT, that is optimized to be used in mental health domains, and improves the accuracy of NLP in easily identifying subtle emotional states using domain specific corpora.

Researchers are tackling AI ethics in mental health, covering embodied AI, digital phenotyping, and risk categorization—addressing privacy, transparency, and equity. Our Adaptive Emotion-Aware Chatbot leverages recurrent RL and transformers for dynamic diagnosis, prioritizing ethical compliance in future work.

Embodied AI (social robots, virtual therapists) has potential to introduce novel treatments and access in mental health (Fiske et al., 2019), though it brings about ethical issues such as data ethics, regulatory lapses, autonomy, transparency, and threats of inequality. The paper evaluates advantages and obstacles in psychiatry/psychology/psychotherapy and calls on research and circumstances on responsible use. Digital phenotyping (Martinez-Martin et al., 2018) is a method of using smartphone/wearable data to passively and objectively assess mental health and is set to grow beyond research into healthcare and consumer applications. It has ethical/legal issues in transparency, informed consent, privacy and accountability because of massive data collection and unprecedented categories of risks. Guidelines should be provided as early as possible in order to exploit its potential without detrimental side effects. The mapping review in (Morley et al. 2020) has classified risks of AI ethics in healthcare as having their epistemic (e.g., unreliable evidence) normative (e.g., unfair outcomes), and traceability aspects, which range between the individual and societal level. It filtered 156 papers to inform policymakers on mindful deployment of AI to save on costs and improve care. The swift action is encouraged to avoid the mistrust of the population and AI winter.

Together, the literature demonstrates a shift toward intelligent, multi-modal, and adaptive mental health assessment systems rather than fixed questionnaires. While ultra-brief screeners such as PHQ-2 and PHQ-DEP-4 reduce burden with a fixed subset of items, they are static and may sacrifice diagnostic information. In contrast, our approach retains the full PHQ-9/GAD-7/PSS-10 item pools and adaptively decides, per user, how many and which items are necessary to reach a reliable decision. Although these innovations can revolutionize the diagnostic accuracy and personalization, their use is dependent on integrity and accuracy of the diagnostic process. Reinforcement learning is used in our proposed model discussed below because the problem addressed here is sequential, partially observable, and multi-objective in nature (accuracy vs. burden), which is hard to encode as fixed rules. The PPO-LSTM agent can learn history-dependent policies over mixed text-derived scores and ROC thresholds, something that is cumbersome for rule-based or classical Bayesian adaptive testing to hand-design for three intertwined disorders and chatbot-style interactions.

## 4 Material and method

The MHP Anxiety, Stress, and Depression dataset is one of the databases that offer a lot of information about the mental health conditions of students, including the rates of anxiety, stress, and depression. The collection of the data was conducted with the help of the popular psychological assessment instruments, i.e., the Generalized Anxiety Disorder 7 items scale (GAD-7), the Perceived Stress Scale 10-item version (PSS-10), and the Patient Health Questionnaire 9-item version (PHQ-9). These standardized scales are widely used in both clinical and research practices to assess the intensity of anxiety, stress, and depressive symptoms. The given dataset includes the responses to the questionnaires above that include the wide range of mental health indicators within the heterogeneous student groups. It is an important source of information about mental health patterns among young adults and especially in terms of academic stress and social influences particular to the group of Bangladeshi students. With its complete and rich collection of responses, this dataset has potential to inform both the public health initiatives and targeted mental health intervention for university students.

Sentiment Analysis for Mental Health dataset is a carefully edited set of statements representing different aspects of mental health with certain attention paid to the emotional and psychological state of a person. The dataset that has more than 51, 000 records and classifies the statements under four different labels; these labels are: Normal, Depression, Stress, and Anxiety. All statements are labeled based on the reflected mental health status, which makes it a crucial tool when training sentiment analysis models. The dataset is quite useful in terms of the textual information that can be processed with the help of natural language processing (NLP) where the aim is to identify mental illnesses using language as a predictor. It can capture several types of utterances, both normal every-day and more painful terms related to depression, anxiety, and stress, providing a more subtle approach to the way people communicate their mental conditions. The researchers and practitioners in fields of psychology, psychiatry and computational linguistics can utilize this dataset to develop algorithms that would automatically detect and analyze the mental health condition based on textual data to establish early detection and intervention in mental health care.

### 4.1 Questionnaire

The questionnaire of the generalized anxiety disorder-7 (GAD-7), perceived stress scale-10 (PSS-10), and patient health questionnaire-9 (PHQ-9) are standardized questionnaires of psychological test, which are self-reported and have question and answer format to assess the level of anxiety, perceived stress, and symptoms of depression. The GAD-7 is a 7-item scale, and all the ratings are made on a four-item Likert scale: 0 = Not at all, 1 = several days, 2 = more than half days and 3 = almost every day. The summed item scores are added to obtain the total score of 0 to 21 and the interpretation is as follows:

- 0–4: Minimal anxiety
- 5–9: Mild anxiety
- 10–14: Moderate anxiety
- 15–21: Severe anxiety.

The PSS-10 consists of ten items rated on a five-point Likert scale (0 = Never to 4 = Very often). Four items (Items 4, 5, 7, and 8) are positively worded and require reverse scoring (0 → 4, 1 → 3, 2 → 2, 3 → 1, 4 → 0). The total score ranges from 0 to 40 and is categorized as:

- 0–13: Low perceived stress
- 14–26: Moderate perceived stress
- 27–40: High perceived stress.

The PHQ-9 consists of nine items assessing depressive symptom frequency over the past 2 weeks, using the same 0–3 scoring metric as the GAD-7. The aggregate score ranges from 0 to 27, with the following severity thresholds:

- 0–4: Minimal depression
- 5–9: Mild depression
- 10–14: Moderate depression
- 15–19: Moderately severe depression
- 20–27: Severe depression.

These instruments are widely validated and enable reproducible, quantitative assessment of mental health symptomatology, facilitating both clinical screening and research-based population studies.

### 4.2 Methodology

The presented research combines multiple advanced components for diagnosing three key mental health disorders: anxiety, stress, and depression. The basis of this diagnostic system is a two-part approach: (1) fine-tuned transformer-based sentiment analysis using RoBERTa (Liu et al., 2019), and (2) reinforcement learning (RL) for dynamic, efficient questionnaire management.

First, the system employs a RoBERTa model. RoBERTa stands for Robustly optimized BERT approach. It is a language model for natural language processing (NLP). Its common applications include text classification (sentiment analysis, spam detection, etc.), question answering, Named Entity Recognition (NER), text generation, etc. In this paper, we have fine-tuned and trained RoBERTa on datasets related to mental health for sentiment analysis. The model provides the probability of the presence of each of the disorders when users provide the text input, providing a quantitative measure of anxiety, stress, depression, and the person not suffering from any of the disorders, i.e., probability of the user being normal. This sentiment analysis step is particularly useful in tackling one significant shortcoming of the conventional ordinal questionnaires—the potential for self-report bias, whereby non-clinical respondents might report underestimating their own state due to unawareness or stigma. Rather than depending exclusively on users' self-reported option scores (e.g., the usual 0–3 values for GAD-7 or PHQ-9 scales), RoBERTa-derived sentiment probabilities are rescaled to the standard scoring range (e.g., 0–4) for objective severity estimation according to clinical standards.

Second, the system formalizes each questionnaire's diagnostic thresholds—the minimum cumulative score at which a disorder can be considered present—by applying Receiver Operating Characteristic (ROC) curve analysis to historical datasets. The Receiver Operating Characteristic (ROC) analysis is an approach that is applied to measure the performance of a binary classification model at each possible classification threshold. ROC curve is a graph which is used to plot the True Positive rate (TPR)/Sensitivity Vs the false positive rate (FPR)/Specificity at different thresholds. It can be employed as a useful method of establishing the optimal classification point of a model. This application is aimed at finding a threshold beyond which the risk of occurrence of the condition is more likely. To enhance predictive accuracy, the ROC analysis identifies a cut off score of each question on the questionnaire that gives the best differentiation between likely true positives and negatives. ROC analysis was conducted separately for each individual question across the three questionnaires using the reference diagnostic outcome. The optimal threshold for each item was determined from the ROC curve by selecting the cut-off that maximized the balance between sensitivity and specificity. Thresholds above this value were interpreted as indicating increased likelihood of the disorder.

The reinforcement learning component [using PPO (Schulman et al., 2017; Huang et al., 2022)] uses the ROC thresholds within an OpenAI Gym-based environment that simulates real assessment sessions. According to the user's current cumulative score and the calculated threshold, the RL agent determines at every stage whether additional questions from a specific questionnaire are necessary. Consequently, the agent can optimize diagnostic efficiency. It can be done without losing accuracy by adaptively eliminating unnecessary queries. The reward system is structured such that correct predictions and essential questions yield positive reinforcement. Moreover, redundant or irrelevant inquiries are penalized, guiding the agent toward optimal questionnaire policies.

Overall, this integrated architecture combines the context-sensitive, human-like language understanding of RoBERTa with data-driven, dynamic decision-making via recurrent RL. It combats subjectivity of self-report mechanisms as well as inefficiency of static questionnaires to allow timely and precise identification of mental health disorders in adaptive and easy to use assessments.

Moving further, to create a dynamic questionnaire, the Recurrent Reinforcement Learning model is used. Reinforcement Learning (RL) is a type of Machine Learning where an agent learns how to make decisions by interacting with the environment. The core idea is, the agent observes the state of the environment and takes action. Based on the action and current state, the environment gives back a reward and a new state. The agent's goal is to learn a policy (a strategy for choosing actions) that maximizes the cumulative long-term score. Mathematically, RL often models problems as a Markov Decision Process (MDP), which assumes the current state contains all the necessary information about the past.

The limitation of the basic RL is the Markov assumption that the agent can see the full state of the environment at each step. But in the real world (and in many simulated tasks), the agent only has partial observations. This is called a Partially Observable MDP (POMDP). So, instead of using a simple feed-forward neural network as the policy/value function, we use a Recurrent Neural Network (RNN) like LSTM or GRU. The RNN has the memory of the past inputs (observations, actions, rewards). This allows the agent to implicitly reconstruct the hidden state of the environment from history. At each time step, the agent gets an observation (not the full state). The RNN maintains a hidden state that summarizes history. The hidden state plus the current observation is used to pick an action. This makes the agent capable of handling partial observability and long-term dependencies.

The dynamic questionnaire system is developed using a recurrent reinforcement learning (RRL) approach, specifically by applying the Proximal Policy Optimization (PPO) algorithm combined with an LSTM-based policy network (MlpLstmPolicy). PPO is a reinforcement learning (RL) algorithm that trains an agent to make optimal decisions in an environment. It's a policy gradient method, which means it learns a policy (a strategy for taking actions) by directly optimizing a function that represents how good that policy is. The recurrent architecture allows the agent to effectively process and leverage the sequential dependency of questions and user responses—crucial for modeling the temporal aspects of adaptive diagnostics in mental health assessments. The training environment for the agent was built using OpenAI's Gym toolkit, and its design reflects several strict requirements: The agent must ask at least one question from each of the three disorder-specific standard questionnaires before proceeding, ensuring comprehensive initial data collection. For certain disorders, such as anxiety, the agent must pose all items from clinically accepted measurement instruments (e.g., the GAD-7), in alignment with standard diagnostic protocols; similar completion rules apply to other disorders under consideration.

The reward system is set up to promote correct and productive diagnostic conduct. Asking the right questions and correctly predicting disorders are the two main behaviors that earn the agent favorable rewards. On the other hand, it is penalized for posing pointless, duplicate, or irrelevant queries, which discourages pointless interactions and promotes diagnostic effectiveness. This framework transforms the task into a partially observable Markov decision process (POMDP), where the agent iteratively decides which question to ask next based on the cumulative answers received, updating its internal representation of the patient's state at each step. Upon reaching predefined diagnostic thresholds for any given disorder, the agent can commit to a prediction, receiving a positive reward if the prediction is accurate.

The overarching objective is to enable the RL agent to learn an optimal policy that balances comprehensive information gathering with minimization of patient burden—in other words, to maximize the accuracy of mental health disorder identification using the fewest possible questions. The recurrent neural network structure (LSTM layers) is vital for maintaining and updating context over the course of the questionnaire, enabling the agent to make informed, history-aware decisions at each stage. The implementation builds upon established frameworks and follows guidelines recommended by the Stable Baselines3 repository for recurrent PPO agents.

**Figure 1:**
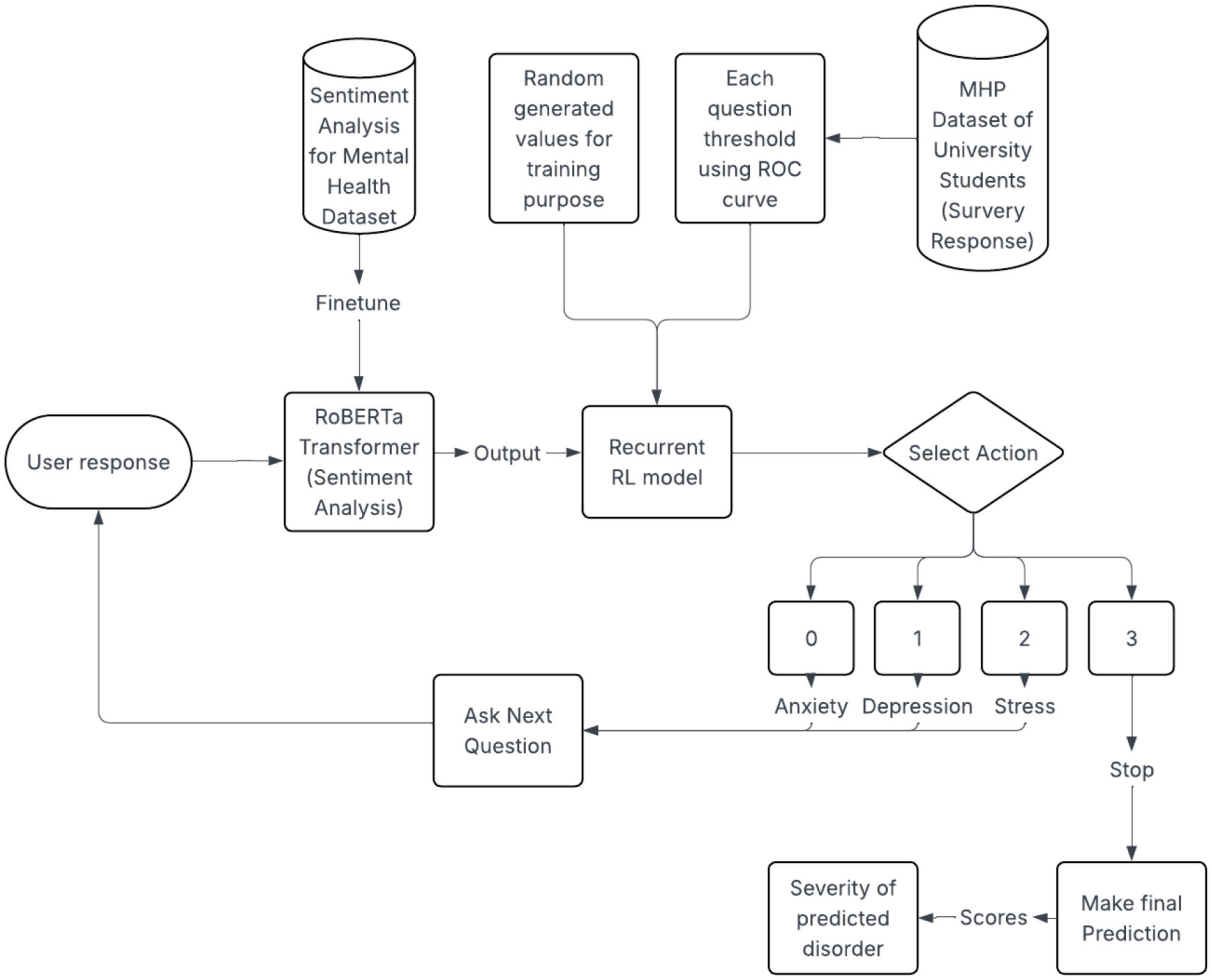
Methodology.

Figure 1 illustrates the flow of the developed model. Our approach begins with fine tuning the RoBERTa transformer model with the dataset. In addition, the RL model is trained on the dataset (Syeed et al., 2024)^1^ along with synthetically generated conversational data. Once the user starts the assessment, the Recurrent RL model begins with asking a question, following which the user will provide a response. This user response is passed through the fine-tuned transformer model which performs sentiment analysis and returns an array which will basically contain scores for each of the three disorders and the score indicating if the person is normal. These scores are passed through the trained Recurrent RL model, which will consider the current as well as the past response scores and return an action. If the action returned is 3, the model will stop and return the results (which is basically the prediction made by the model of whether the person is suffering with one or more mental health disorders or has none of it). If the action returned is one of the values from 0, 1 or 2, the model will move forward to ask the next question from the respective category (0—for anxiety, 1—for depression and 2— for stress). The scores used in system are used as a technical intermediate representation that is aligned to GAD-7/PHQ-9/PSS-10 thresholds.

#### 4.2.1 Observations and RoBERTa

At time *t*, if the agent asks a question (*a*_*t*_ ∈ {0, 1, 2} the user replies with text *x*_*t*_. RoBERTa (fine-tuned classifier) maps *x*_*t*_ to a score vector:


$$o_{t}=f_{\text{RoBERTa}}\left(x_{t}\right)=(o_{t}^{A},o_{t}^{D},o_{t}^{S},o_{t}^{N})\in {\mathbb{R}}^{4}.$$

If the agent chooses Stop (*a*_*t*_=3), there is no new user reply and the observation will be a last observation repeated.

Define discrete question offsets / counters per disorder:

- $q_{t}^{A}$ = index of next Anxiety question to ask (or number asked so far),
- similarly $q_{t}^{D},q_{t}^{S}$.

Define cumulative scores:


$$S_{t}^{A}=\underset{k\in T_{A}\left(t\right)}{\sum }o_{k}^{A}\;S_{t}^{D}=\underset{k\in T_{D}\left(t\right)}{\sum }o_{k}^{D}\;S_{t}^{S}=\underset{k\in T_{S}\left(t\right)}{\sum }o_{k}^{S}$$

where *T*_*A*_(*t*) is the set of timesteps up to *t* where Anxiety questions were asked (similarly for Depression and Stress).

#### 4.2.2 Problem as a POMDP

Because the agent doesn't see the true latent user mental state but only RoBERTa scores (and history), model this as a Partially Observable Markov Decision Process (POMDP) defined by the tuple


M=(S,A,T,R,Z,O,γ)

Where:

- S: state of the user.
- A={0,1,2,3}: discrete actions (0 = ask Anxiety, 1 = ask Depression, 2 = ask Stress, 3 = Stop)
- $O\subset {\mathbb{R}}^{4}$: observation space. Each observation is an array of four scores from RoBERTa:

$$\left(o_{t}^{A},o_{t}^{D},o_{t}^{S},o_{t}^{N}\right).$$

- *T*(*s*_*t*+1_∣*s*_*t*_, *a*_*t*_): transition on true user state.
- *Z*(*o*_*t*_∣*s*_*t*_, *a*_*t*−1_): observation likelihood (RoBERTa mapping user reply → scores).
- R: reward function
- γ ∈ [0, 1): discount factor.

An episode *e* runs until the agent chooses action 3 (Stop) and the environment allows termination (see reward rules).

#### 4.2.3 Reward function

Let final thresholds be τ^*A*^, τ^*D*^, τ^*S*^. Let $Q_{\max}^{A},Q_{\max}^{D},Q_{\max}^{S}$ be the number of questions available per disorder. Let an indicator ${asked}_{t}^{A}$ denote whether all Anxiety questions have been asked up to time *t* (i.e., $q_{t}^{A}=Q_{\max}^{A}$). Similar for ${asked}_{t}^{D},{asked}_{t}^{S}$.

For action *a*_*t*_:

Case *a*_*t*_=0 (ask Anxiety):


$$R_{t}=\{\begin{array}{ll} -5 & {\text{if asked}}_{t}^{A}\;(\text{Decision already taken for Anxiety})\\ +10 & \text{if }({\text{asked}}_{t}^{A})\wedge (S_{t}^{A}\geq \tau^{A})\;(\text{Anxiety predicted})\\ -1 & \text{if }(\neg {\text{asked}}_{t}^{A})\wedge (q_{t}^{A}<Q_{\max^{A}}\;\vee \;S_{t}^{A}>\theta_{q_{t}^{A}}^{A})\\ -5 & \text{otherwise.} \end{array}$$

Here $\theta_{q_{t}^{A}}^{A}$ denotes the per-question dynamic threshold you described (“current question threshold”). (Replace analogous variables for Depression and Stress.)

Case *a*_*t*_=1 (ask Depression)—same pattern with *S*^*D*^, τ^*D*^, *q*^*D*^, θ^*D*^.

Case *a*_*t*_=2 (ask Stress)—same pattern with *S*^*S*^, τ^*S*^, *q*^*S*^, θ^*S*^.

Case *a*_*t*_=3 (Stop):

Define


$$\begin{array}{l} \;({\text{asked}}_{\text{t}}^{\text{A}}\;\vee \;{\text{S}}_{\text{t}}^{\text{A}}\leq \theta_{{\text{q}}_{\text{t}}^{\text{A}}}^{\text{A}})\;\wedge \\ \text{stop}\_{\text{ok}}_{\text{t}}\equiv ({\text{asked}}_{\text{t}}^{\text{D}}\;\vee \;{\text{S}}_{\text{t}}^{\text{D}}\leq \theta_{{\text{q}}_{\text{t}}^{\text{D}}}^{\text{D}})\;\wedge \\ \;({\text{asked}}_{\text{t}}^{\text{S}}\;\vee \;{\text{S}}_{\text{t}}^{\text{S}}\leq \theta_{{\text{q}}_{\text{t}}^{\text{S}}}^{\text{S}}) \end{array}$$

(Equivalently: stop is allowed only if none of the unpredicted disorders require more questions by the rules given.)


$$R_{t}=\{\begin{array}{cc} -5, & \begin{array}{l} \text{if any disorder not yet predicted and its questions are }\\ \text{ remaining or its score exceeds its current threshold }\\ \;(\text{i}.\text{e}.,stop\_ok_{t}=\;False). \end{array}\\ +20, & \begin{array}{l} \text{if }stop\_ok_{t}=True\;(\text{generate final result and}\\ \text{terminate}) \end{array} \end{array}$$

A penalty of –5 is returned if none of the disorders is predicted and no questions from its questionnaire are asked, or if its score exceeds its current threshold (both of which basically mean that, according to the rules defined, questions still need to be asked). Additionally, upon termination, the final result will be returned, which will basically give the diagnosis made by the RRL model.

#### 4.2.4 Termination and episode handling

Episode ends at earliest time *T* such that agent executes action 3 and *stop*_*ok*_*T*_ = True. The policy must learn to choose Stop only when validated by the reward (i.e., +20 shown); otherwise Stop yields a heavy penalty −5.

#### 4.2.5 Summary of variables/notation

- $o_{t}=\left\{o_{t}^{A},o_{t}^{D},o_{t}^{S},o_{t}^{N}\right\}$ : RoBERTa observation vector at time *t*.
- $S_{t}^{A},S_{t}^{D},S_{t}^{S}$ : cumulative disorder scores.
- $\theta_{q}^{A},\theta_{q}^{D},\theta_{q}^{S}$ : per-question thresholds.
- $Q_{\max}^{A},Q_{\max}^{D},Q_{\max}^{S}$ : maximum questions per disorder.
- *a*_*t*_ ∈ {0, 1, 2, 3} : agent action.

The suggested adaptive questionnaire system is quite different in nature to the current existing RL-based dynamic questionnaire systems. In this the clinical questionnaires and ROC-based thresholds are formulated directly as part of the RL problem, rather than regarding question selection as a generic survey optimization. It works directly on GAD-7, PHQ-9, and PSS-10, where ROC-derived per-question thresholds are used to control the RL actions and stopping rule, rather than just minimizing abstract survey length. It as well translates free-text with RoBERTa into Likert-style scores to feed the RL agent to diagnose multiple disorders with just necessary amount of, clinically relevant questions.

#### 4.2.6 Separation of convergence of policies and diagnostic validity

Although the reinforcement learning measures, like cumulative reward, episode length give an insight as to policy learning and convergence behavior, they alone do not determine clinical diagnostic correctness. According to the offered system, the RL agent optimizes the item selection and ending of the questionnaires according to ROC-based thresholds, as well as cumulative scores of severity. Thus, one should make a difference between:

- Policy Convergence—does the agent learn an efficient and constant questioning strategy.
- Diagnostic Validity—the alignment between the final predicted disorder and the clinically established questionnaire thresholds.

The reward function is created to promote the rule compliance to the approved questionnaire. Only in the case of termination based on satisfying disorder-specific threshold criteria, positive reward is awarded. Negative reward is given on early termination or unneeded enquiry. Therefore, reward convergence is an indication of stabilization of rule consistent diagnostic behavior and not arbitrary termination of an episode. But, as reward is an internal optimization signal further statistical analysis of the final diagnostic results is carried out to assess the model's predictive consistency and reliability. This will assure that the assessment has both behavioral and diagnostic competence with the validated measures.

## 5 Results

To refine diagnostic decisions during the assessment process, ROC (Receiver Operating Characteristic) curve analysis is applied to historical datasets. This method helps determine the optimal threshold cumulative score. In this, at each step of the questionnaire, estimating the likelihood. At any intermediate stage, the cumulative score is compared against the corresponding threshold. These thresholds have proved important for training reinforcement learning (RL) models. It ensures efficiency and gives a personalized diagnosis without any hassle. The RoBERTa was fine-tuned with an accuracy of 92.62%. It is used for sentiment analysis, which in turn helps determine the severity of the disorder. For each response, it produces a 4-dimensional output vector with each dimension representing severity of a specific class. The highest-scoring output was used to get the predicted class by comparing it with the ground truth to obtain computer accuracy. This is to make sure that the evaluation of the model correctly captures its potential to place the highest probability on the correct class all the time.

### 5.1 Mean reward (μ_*R*_)

The mean reward, denoted as μ_*R*_, represents the average total reward obtained per episode over a given set of runs. It is calculated as:


$$\mu_{R}=\frac{1}{N}\overset{N}{\underset{i=1}{\sum }}R_{i}$$

where *N* is the total number of episodes, and *R*_*i*_ is the total reward in the *i*^*th*^ episode. This is one of the major signs of the overall performance of the agent in reinforcement learning. An ever-increasing average reward throughout training is also a good sign that the policy is getting better, whereas a plateau could be an indication of convergence. Sudden falls may be an indicator of a lack of stability or a catastrophe of forgetting.

### 5.2 Standard reward (σ_*R*_)

The standard reward, or more formally, the standard deviation of rewards, is denoted as σ_*R*_ and measures the variability in rewards across episodes. It is computed as:


$$\sigma_{R}=\sqrt{\frac{1}{N}\overset{N}{\underset{i=1}{\sum }}{\left(R_{i}-\mu_{R}\right)}^{2}}$$

where μ_*R*_ is the mean reward. This metric indicates how predictable performance of the agent is - low σ_*R*_ indicates that the agent acts in the same way in all the episodes and high σ_*R*_ indicates unpredictability or unstableness. A policy with a low variance (that is, a low variance policy) is more valuable in a safety-critical system than a policy with a marginally larger mean reward.

### 5.3 Minimum reward (*R*_*min*_)

The minimum reward, *R*_*min*_, indicates the worst-case performance in a single episode. It is defined as:


$$R_{\min}=\underset{1\leq i\leq N}{\min}R_{i}$$

and provides insight into the most severe failures the agent can experience. In tasks where catastrophic failure is costly, keeping *R*_*min*_ at a reasonably high level can be as important as maximizing the mean reward. A large gap between μ_*R*_ and *R*_*min*_ might signal occasional but significant failures.

**Minimum Reward (Excluding the outliers)**

Using the box plots, the minimum rewards, excluding outliers, were observed. The box-plot charts, along with the observed values, are presented below.

### 5.4 Maximum reward (*R*_*max*_)

The maximum reward, *R*_*max*_, represents the best performance the agent has achieved in any episode and is given by:


$$R_{\max}=\underset{1\leq i\leq N}{\max}R_{i}$$

While this value shows the upper bound of the agent's capability, it must be interpreted with caution—a high *R*_*max*_ alongside a low μ_*R*_ suggests that good performance is rare and possibly luck-driven, rather than the result of a stable policy.

### 5.5 Mean episode length (μ_*T*_)

The mean episode length, μ_*T*_, is the average number of steps per episode and is calculated as:


$$\mu_{T}=\frac{1}{N}\overset{N}{\underset{i=1}{\sum }}T_{i}$$

where *T*_*i*_ is the length of episode *i*. This metric is very dependent on the task to be performed. In environments that involve survival, the mean episode length can be a longer value, which means the successful evasion of failure states by the agent. On the other hand, in goal-oriented environments shorter episode lengths may be preferable because it indicates agent is trying to achieve the goals faster.

### 5.6 Standard episode length (σ_*T*_)

Finally, the standard episode length, σ_*T*_, measures the variability in episode durations:


$$\sigma_{T}=\sqrt{\frac{1}{N}\overset{N}{\underset{i=1}{\sum }}{\left(T_{i}-\mu_{T}\right)}^{2}}$$

A small σ_*T*_ suggests that episodes are of the same length, which may indicate that the agent pursues a strategy. High σ_*T*_ may signal the unpredictable results—perhaps because of the presence of stochastic environments, exploration, or volatile policies.

Table 1 depicts the performance of the agent across different episodes. To further examine the stability and consistency of these results, the agent's performance was evaluated at episode intervals of 100, 1,000, 5,000, 10,000, and 20,000. Through the comparison between the mean rewards, standard deviations, and episode lengths in these evaluation windows, we are able to see how the statistical estimates converged as the sample size became larger. Smaller evaluation windows may exhibit higher variance due to limited sampling, while larger windows tend to provide more stable and reliable estimates of the agent's true performance distribution.

**Table 1:** RL model evaluation based on the above formulae.

Evaluation metric	Number of episodes				
	**100**	**1,000**	**5000**	**10,000**	**20,000**
Mean Reward	–86.31	–97.68	–102.32	–102.98	–102.96
Standard Reward	65.54	66.45	75.34	76.21	76.79
Minimum Reward	–288.00	–482.00	–816.00	–735.00	–1081.00
Maximum Reward	14.00	14.00	14.00	14.00	14.00
Mean Episode Length	23.80	26.11	27.15	27.33	27.36
Standard Episode Length	13.66	13.71	15.73	15.96	16.12

### 5.7 Histogram interpretation

**Figure 2:**
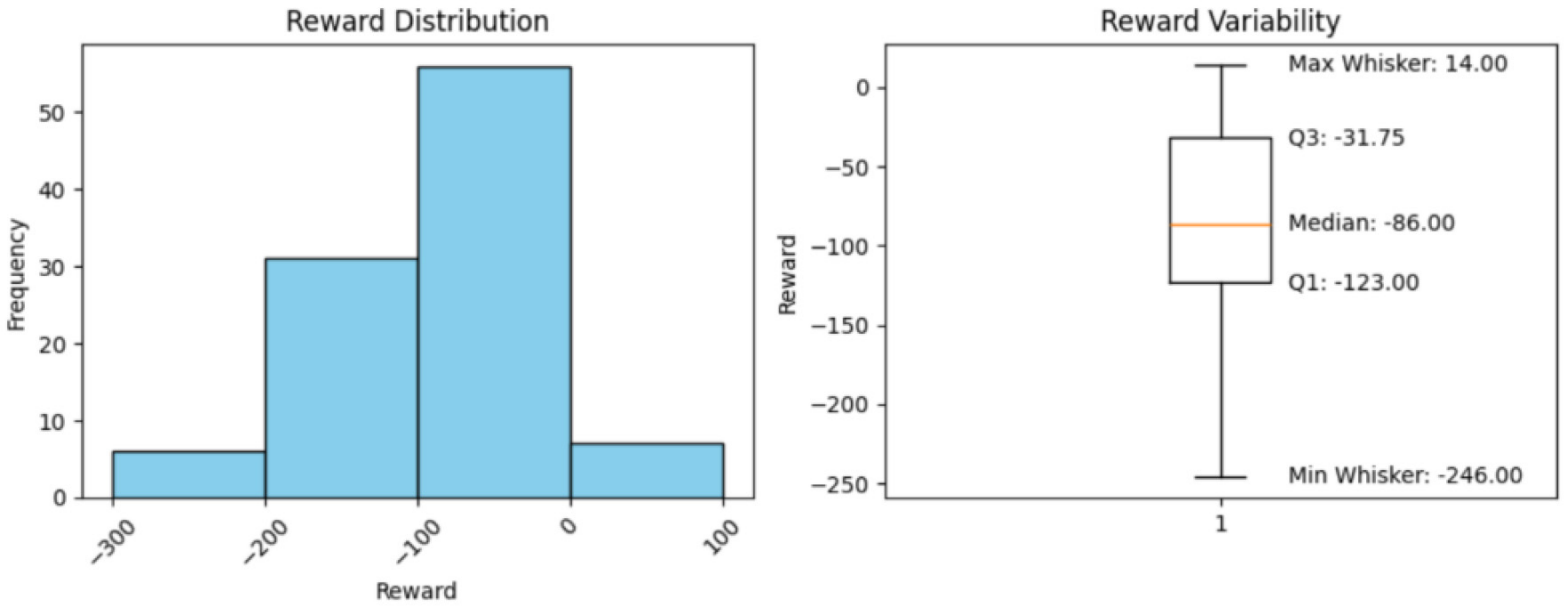
Model performance over 100 steps.

**Figure 3:**
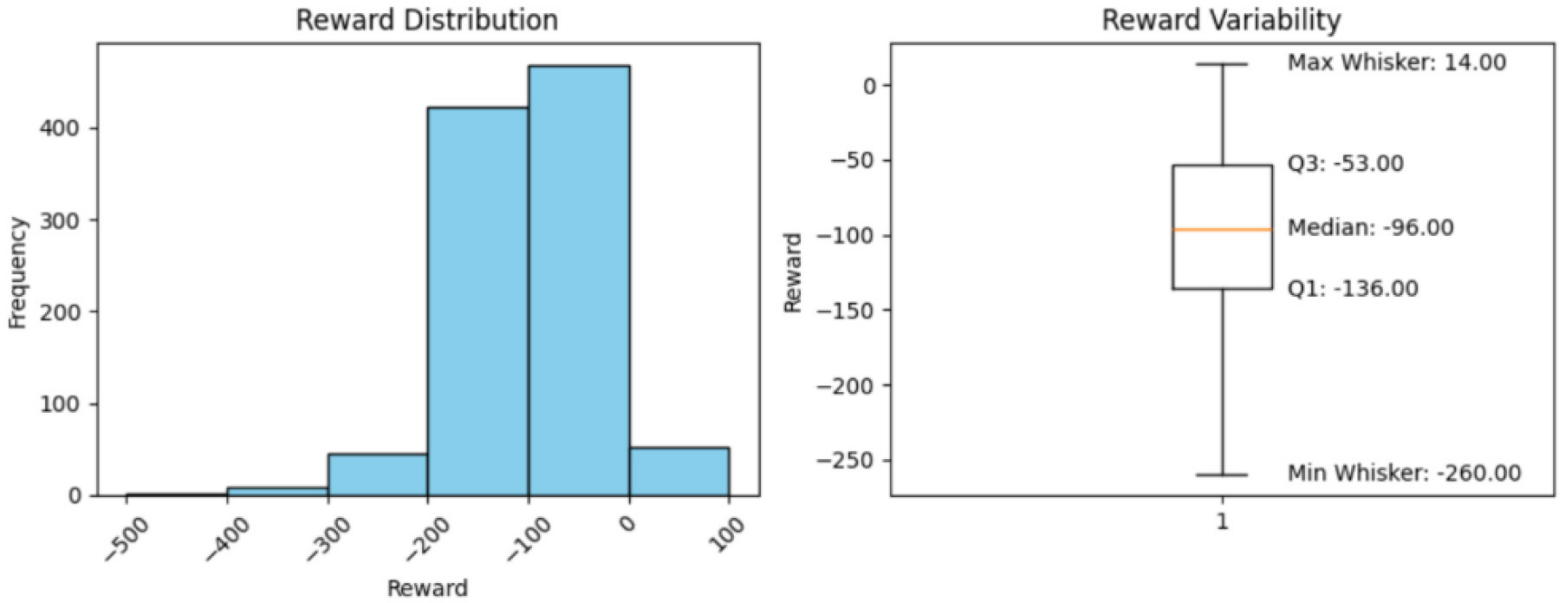
Model performance over 1,000 steps.

**Figure 4:**
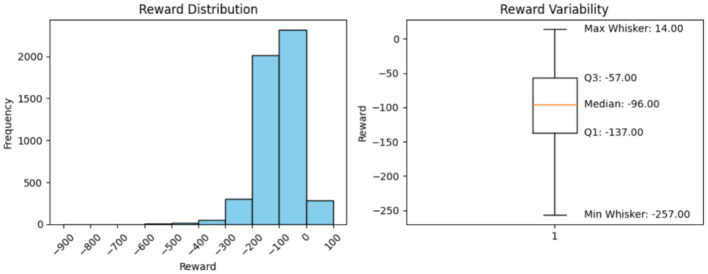
Model performance over 5,000 steps.

**Figure 5:**
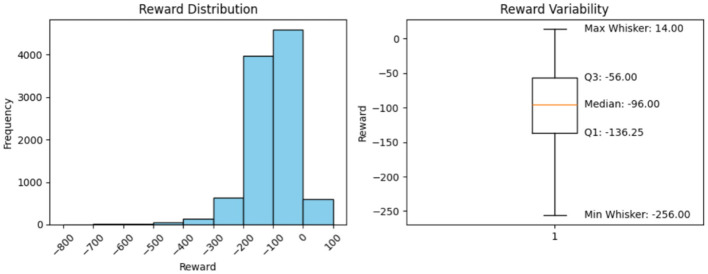
Model performance over 10,000 steps.

**Figure 6:**
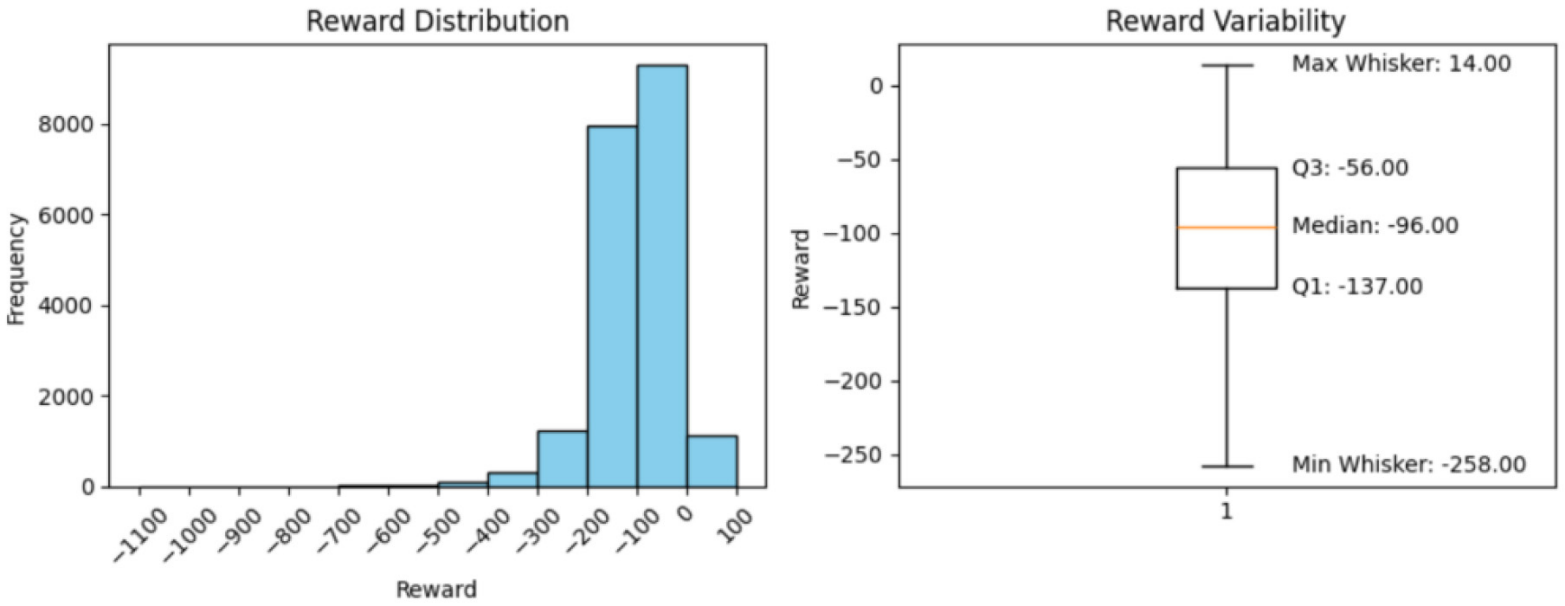
Model performance over 20,000 steps.

The histograms in Figures 2–6 illustrate illustrates the frequency of different rewards. Most rewards for all the above cases fall within the range of –200 to 0, with a higher concentration between –100 and 0.

### 5.8 Box-plot interpretation

Mean, minimum, and maximum rewards can also be obtained from the box plots. The difference between the ones we have found using the formulae and the ones shown by the box plots in Figures 2–6 is that the box plot excludes outliers during calculation, providing a better overview of how the model works. It can be clearly seen from the box plots obtained from 1,000, 5,000, 10,000, and 20,000 episodes that the mean reward remains consistent at a value of –96.0, indicating that the model exhibits good and consistent performance. The maximum reward for all the experiments remains 14.0 while the minimum reward ranges from –260 to –255.

The reward structure includes a per-step penalty to encourage shorter diagnostic sequences and penalties for incorrect or unnecessary actions. A positive reward is granted only upon correct final diagnosis. Due to the accumulation of step-wise penalties and sparse terminal rewards, cumulative episode rewards tend to skew negative.

The histograms in Figures 2–6 reflect cumulative rewards across multiple simulated episodes. For this analysis, episodes were generated using randomly sampled inputs within the defined state space to assess overall behavioral trends.

### 5.9 Statistical analysis of diagnostic stability

The above representation of the reinforcement learning results indicates that the questioning policy has been stabilized. However, convergence of policies does not always mean diagnostic reliability. Thus, further examination was done on the eventual diagnostic results that were achieved at the end of the episode.

The agent generates a final diagnostic decision at the end of every episode, depending on the severity scores that have been accumulated and the pre-determined questionnaire thresholds. The descriptive statistical measures were calculated to determine the consistency of these decisions on the end outputs.

### 5.10 Stability checking using boxplot

**Figure 7:**
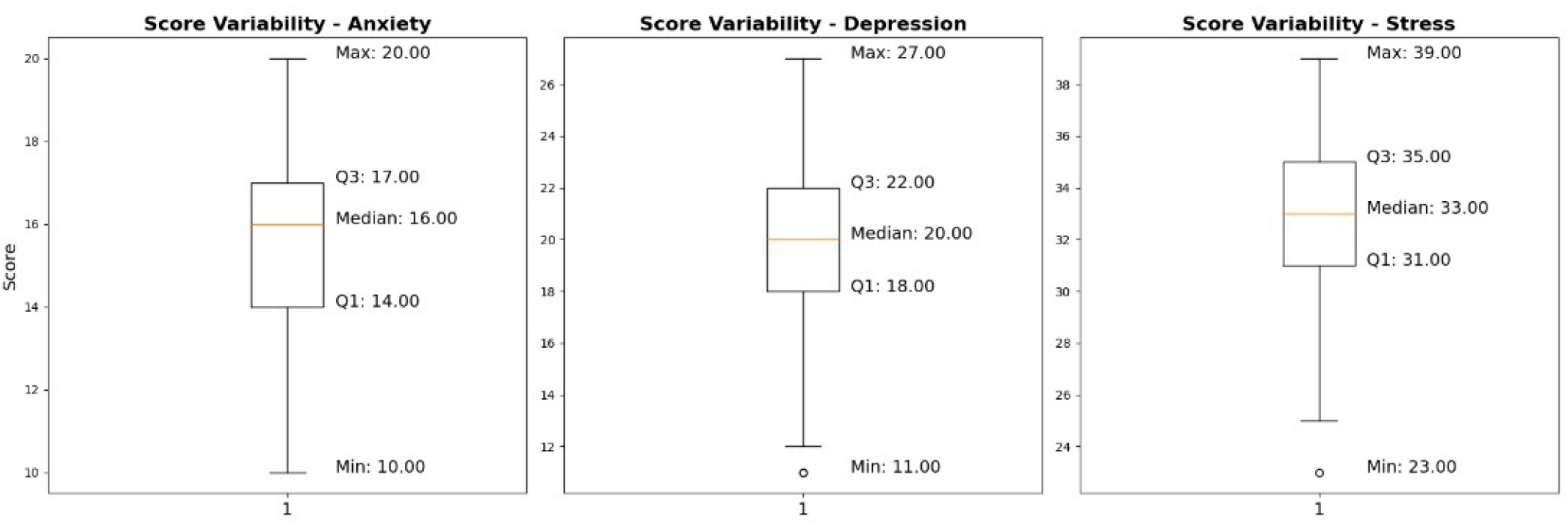
Boxplots of diagnostic output distribution.

Boxplots in Figure 7 were built to further consider the distribution of the final diagnostic results. The boxplot summarizes:

- Median value
- Interquartile Range (IQR = Q3 – Q1)
- Co-existence of extreme outliers.

A short interquartile range shows low variability of diagnostic outcomes. Congruence of median and mean indicates the lack of a strong skewness. The number of extreme outliers is limited, which means that the model does not often create outliers and conflicting diagnostic choices.

The above distributional properties reveal that the trained policy not only stabilizes in reward space but also approaches the same diagnostic output that is consistent across predictive clinical thresholds.

To summarize this quantitatively, Table 2 is a representation of the descriptive statistics in terms of median, min, and max.

**Table 2:** Descriptive statistics of diagnostic outputs.

Disorder	Min	Max	Median
Anxiety	10	20	16
Depression	11	27	20
Stress	23	39	33

### 5.11 Comparison to non-RL baselines

In order to compare the suggested RL-based adaptive framework with non-RL baselines, we looked at its predictions in comparison with complete static questionnaire classifications of every disorder. In particular, the full GAD-7, PHQ-9, and PSS-10 were used as references to anxiety, depression, and stress, respectively.

The standard severity thresholds as per the guidelines of the respective instruments were used. Moderate and above severity was taken as determined for anxiety and depression, and severe and above was taken as determined for stress. These baseline classifications were compared to RL model predictions through confusion matrix analysis and conventional performance measures.

The findings show that there is a good consistency between adaptive RL framework and the related full questionnaires that are purely static, which shows that the proposed methodology does not modify the diagnostic behavior but minimizes the burden of the questions.

The nearly perfect precision and specificity values observed in Table 3 were carefully re-evaluated to exclude potential data leakage or implementation errors. The dataset used in this study was synthetically generated in Python based on domain knowledge of how realistic response patterns should appear. Due to the rule-based and structured nature of the generation process, the resulting class distributions were highly separable, leading to minimal false positives and consequently nearly perfect precision and specificity. These results therefore reflect the controlled characteristics of the synthetic dataset rather than guaranteed real-world generalization.

**Table 3:** Comparative diagnostic performance of RL model against standardized questionnaire baselines.

Disorder	Accuracy	Precision	Recall	F1 score	Specificity
Anxiety	78.90%	100.00%	69.46%	81.98%	100.00%
Depression	81.20%	100.00%	72.51%	84.07%	100.00%
Stress	89.60%	99.19%	70.52%	82.43%	99.69%

**Figure 8:**
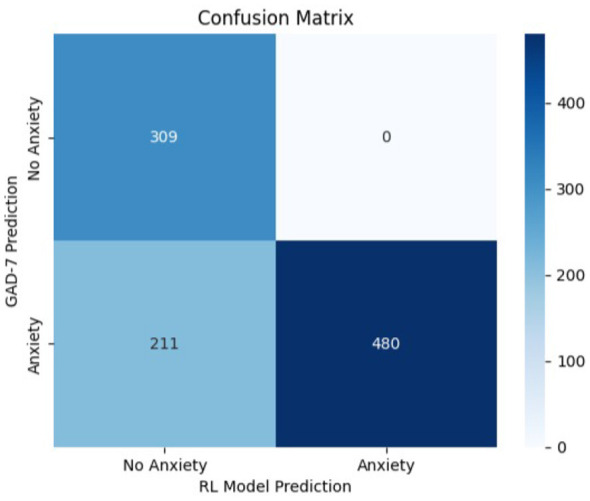
Confusion matrix—RL model vs. GAD-7 baseline (anxiety detection).

**Figure 9:**
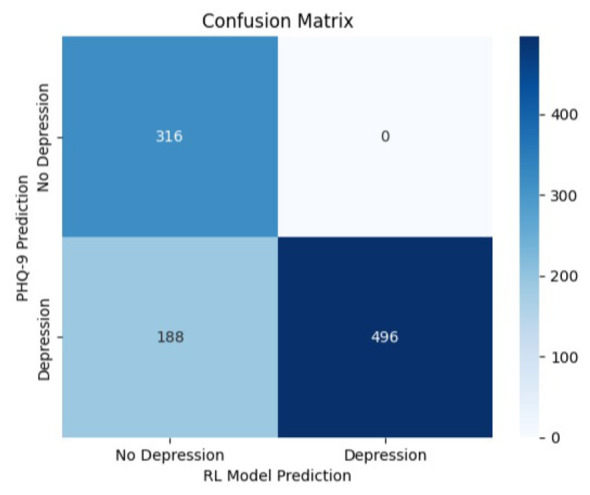
Confusion matrix—RL model vs. PHQ-9 baseline (depression detection).

**Figure 10:**
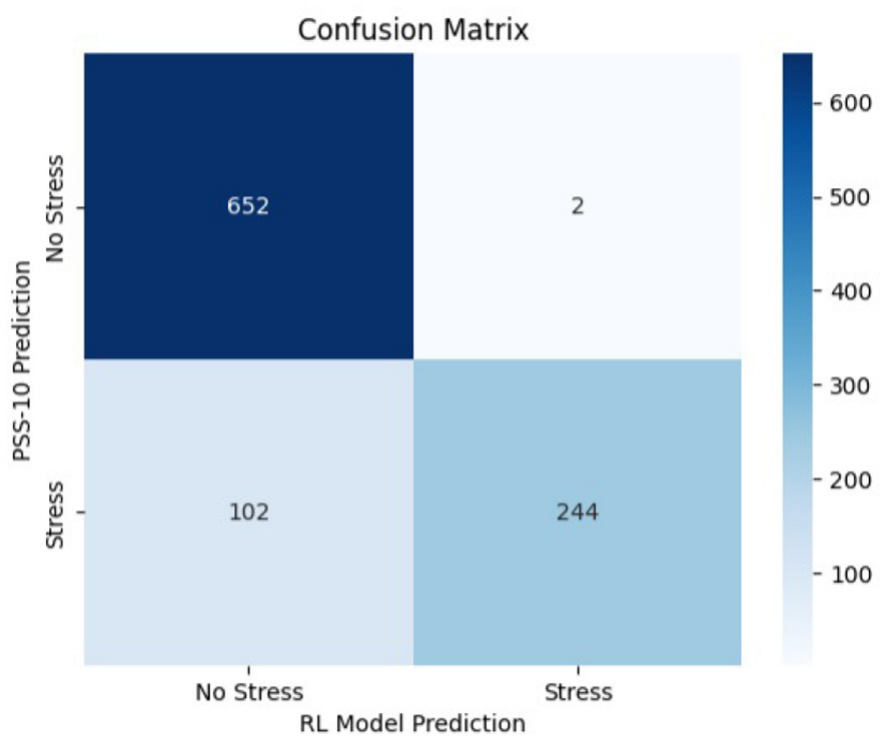
Confusion matrix—RL model vs. PSS-10 baseline (stress detection).

The confusion matrices presented in Figures 8–10 are computed using same independently generated synthetic test set comprising 1,000 samples. These samples were produced using the same structured generation framework without overlap with the training data.

## 6 Conclusion

In this study, we integrated questions from standard assessment tools, such as the GAD-7, PHQ-9, and PSS-10 questionnaires, to develop an emotion-aware chatbot that dynamically adjusts its questioning strategy based on the user's responses. As opposed to non-interactive short-answer assessments like PHQ-2, PHQ-DEP-4, the proposed ROC- and RL-led framework does not make changes and/or incur a decrease in the validated questionnaires. Rather, it chooses to administer either full PHQ-9, GAD-7 or PSS-10 scale selectively depending on user responses, thus ensuring that it maintains diagnostic integrity of the original measures without unrelated questioning. This is an adaptive strategy that is intended to enhance diagnostic performance but retain conversational meaning. To classify the severity, a fine-tuned RoBERTa model attained the accuracy of 92.62%. The ROC curve analysis was also conducted on every question to identify the best threshold scores to have an accurate map between user response and level of severity. These thresholds explicitly control the decision making process of the reinforcement learning (RL) agent on the next question to ask. The RL model also demonstrated the desired adaptive questioning behavior whereby items with a high level of diagnostic value were given priority, and unnecessary repetitions were minimized. The dynamic termination of questioning through the use of custom POMDP environment of ROC-derived per-question thresholds allows the recurrent PPO-LSTM agent to competently avoid redundant queries by terminating the questioning process at disorder-specific cutoffs with a fine-tuned reward function. Clustering the RL model with the classification output of RoBERTa and ROC-based thresholds played an important role in the model performance. Although the system was designed successfully, it could be improved further by designing a better structure of the environment, including more various types of conversational states and modifying the reward function by balancing accuracy with user engagement. Furthermore, the system is more to be used as a screening and decision-support device and not a substitute to professional clinical assessment. Although it aids in initial risk recognition, end diagnosis and treatment is the task of the qualified medical staff.

## 7 Limitations

- The chosen ROC thresholds were not manually or externally checked and were data based. Thus, other choices of cut-off can have an impact on the sensitivity-specificity trade-off and change the results of classification.
- Another part of this work in the future is systematic assessment and enhancement of the RoBERTa model to be less susceptible to sarcastic, ambiguous, and culturally specific responses with the help of specific test sets and human-in-the-loop verification.
- Since the model was trained using data that was synthetically generated, its performance might not be able to reflect the range of human conversations in the real world.
- The current analysis is only on the population of the students in the MHP data. In this regard, the results can not necessarily be extrapolated to other population segments. The framework, however, is flexible and can be reconfigured with population-specific data and ROC thresholds to have a greater applicability.
- Although the proposed model achieved nearly perfect precision and specificity on the synthetic dataset, such performance is influenced by the structured and rule-based data generation process. Real-world datasets typically contain greater variability, ambiguity, and noise. Future work will involve evaluating the model on independently collected real-world data to further assess robustness and generalizability.

## Code availability

The complete codebase, including the synthetic data generation scripts and the final dataset values used in the experiments, is publicly available at: text footnote ^2^. This ensures full reproducibility of the training, testing, and evaluation procedures.

## Data Availability

The original contributions presented in the study are included in the article/supplementary material, further inquiries can be directed to the corresponding author.

## Footnotes

1 https://www.kaggle.com/datasets/suchintikasarkar/sentiment-analysis-for-mental-health - Sentiment Analysis for Mental Health Dataset

2
https://github.com/soniadessai/Mental-Health-Diagnosis-Using-Reinforcement-Learning
